# Brevenal Inhibits Pacific Ciguatoxin-1B-Induced Neurosecretion from Bovine Chromaffin Cells

**DOI:** 10.1371/journal.pone.0003448

**Published:** 2008-10-20

**Authors:** César Mattei, Peter J. Wen, Truong D. Nguyen-Huu, Martha Alvarez, Evelyne Benoit, Andrea J. Bourdelais, Richard J. Lewis, Daniel G. Baden, Jordi Molgó, Frédéric A. Meunier

**Affiliations:** 1 CNRS, Institut de Neurobiologie Alfred Fessard, Laboratoire de Neurobiologie Cellulaire et Moléculaire, Gif-sur-Yvette, France; 2 Queensland Brain Institute and School of Biomedical Sciences, The University of Queensland, Brisbane, Australia; 3 Photonics and Mathematical Optics Group, Tecnológico de Monterrey, Monterrey, México; 4 Center for Marine Science, University of North Carolina at Wilmington, Wilmington, North Carolina, United States of America; 5 Institute for Molecular Bioscience, The University of Queensland, Brisbane, Australia; National Institutes of Health, United States of America

## Abstract

Ciguatoxins and brevetoxins are neurotoxic cyclic polyether compounds produced by dinoflagellates, which are responsible for ciguatera and neurotoxic shellfish poisoning (NSP) respectively. Recently, brevenal, a natural compound was found to specifically inhibit brevetoxin action and to have a beneficial effect in NSP. Considering that brevetoxin and ciguatoxin specifically activate voltage-sensitive Na^+^ channels through the same binding site, brevenal has therefore a good potential for the treatment of ciguatera. Pacific ciguatoxin-1B (P-CTX-1B) activates voltage-sensitive Na^+^ channels and promotes an increase in neurotransmitter release believed to underpin the symptoms associated with ciguatera. However, the mechanism through which slow Na^+^ influx promotes neurosecretion is not fully understood. In the present study, we used chromaffin cells as a model to reconstitute the sequence of events culminating in ciguatoxin-evoked neurosecretion. We show that P-CTX-1B induces a tetrodotoxin-sensitive rise in intracellular Na^+^, closely followed by an increase in cytosolic Ca^2+^ responsible for promoting SNARE-dependent catecholamine secretion. Our results reveal that brevenal and β-naphtoyl-brevetoxin prevent P-CTX-1B secretagogue activity without affecting nicotine or barium-induced catecholamine secretion. Brevenal is therefore a potent inhibitor of ciguatoxin-induced neurotoxic effect and a potential treatment for ciguatera.

## Introduction

Ciguatera is the most widespread human syndrome of marine poisoning that occurs following consumption of fish from tropical and subtropical pacific regions contaminated with ciguatoxins that accumulate through the marine food chain [1]. It is mainly characterised by neurological, gastrointestinal and cardiovascular disturbances [2]. Ciguatera is a significant public heath issue in endemic areas with up to 50,000 cases recorded annually [3]. Importantly, it is becoming a widespread health problem with the globalisation of the fishing industry importing ciguateric fish from endemic regions [3]. No specific treatment other than symptomatic treatment is currently available for this disease. Interestingly, research with the toxic dinoflagellate that produces brevetoxins, *Karenia brevis*, has resulted in the discovery of natural and chemically modified compounds that may have the potential for the treatment of ciguatera.

Ciguatoxins and brevetoxins are lipid-soluble cyclic polyether compounds produced by the benthic dinoflagellates *Gambierdiscus toxicus* and *Karenia brevis*, respectively, which bind competitively to the receptor-site 5 of voltage-sensitive Na^+^ channels [4], [5], [6]. One of the well-established consequences of ciguatoxins' action is an increase in membrane Na^+^ permeability and excitability of most excitable cells [7]. In particular, Pacific ciguatoxin-1B (P-CTX-1B previously called CTX and CTX-1b) has been shown to enhance voltage-sensitive Na^+^ channels activity by shifting their activation to more negative membrane potentials, and by inhibiting in part their inactivation [8]. At the frog neuromuscular junction, P-CTX-1B was reported to induce repetitive endplate potentials that elicit repetitive muscle action potentials. In addition, this toxin causes a dramatic increase in asynchronous acetylcholine release and impairs synaptic vesicle recycling [9]. These effects are believed to play an important role in the development of ciguatera disease [10]. Very little is known about how Na^+^ channel activation leads to asynchronous quantal spontaneous release. One of the possible hypotheses is that ciguatoxins promote Ca^2+^ entry in nerve terminals, which could in turn stimulate exocytosis. Importantly, the activation of Na^+^ channels by P-CTX-1B has been shown to lead to direct mobilisation of Ca^2+^ from internal stores in neuroblastoma cells and rat myotubes [11], [12] and to an indirect activation of the Na^+^/Ca^2+^ exchanger in the reversed mode in cholinergic synaptosomes, allowing Ca^2+^ influx against Na^+^ efflux [13].

In this study, we have clarified the role played by ciguatoxin-induced long lasting Na^+^ influx in promoting Ca^2+^-dependent neuroexocytosis, by assessing the effect of P-CTX-1B on neuroendocrine cells. Chromaffin cells are a convenient model to study both Ca^2+^ and Na^+^ signalling together with catecholamine release [14]. Having reconstituted the sequence of events leading to neuronal secretion, we used newly-developed competitive inhibitors of brevetoxin to test whether they were able to prevent ciguatoxin-induced secretagogue activity. We found that both brevenal and β-naphtoyl-brevetoxin are potent inhibitors of P-CTX-1B-induced catecholamine secretion without affecting the response to other secretagogues.

## Results

### P-CTX-1B promotes a slow cytosolic Na^+^ increase in chromaffin cells

**Figure 1:**
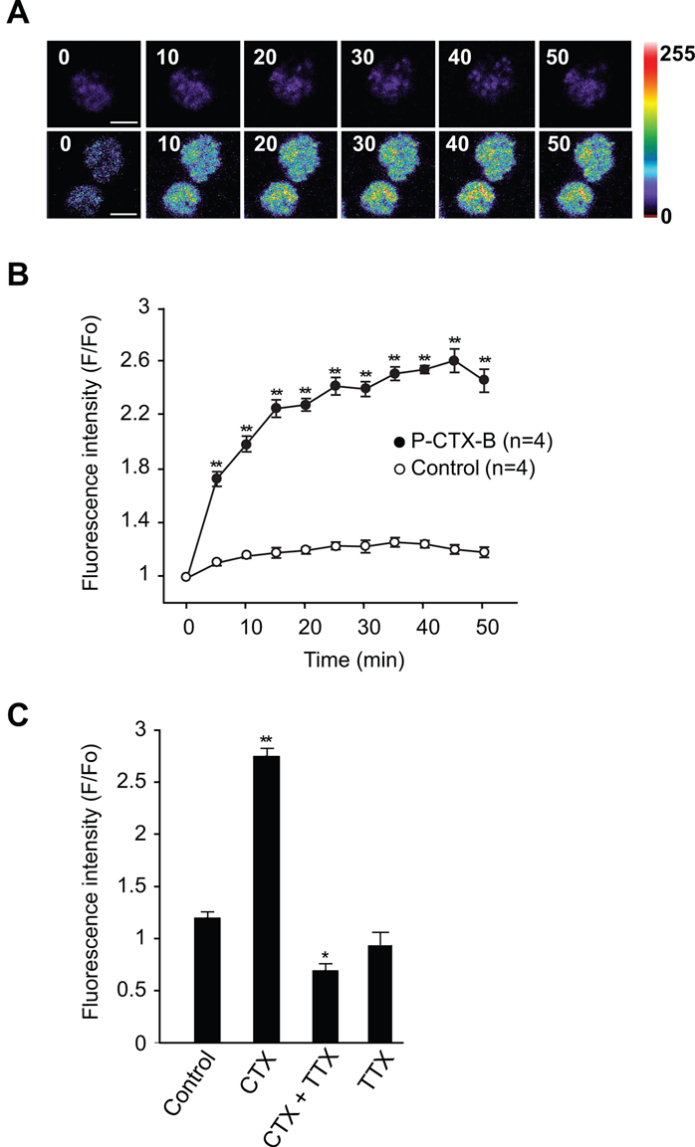
P-CTX-1B induces sodium influx into chromaffin cells. Chromaffin cells loaded with cell permeable sodium-green (6 µM) supplemented with 0.01% of pluronic acid for 45 min and washed before examination by time-lapse confocal microscopy at 5 min/frame. (A): Sodium-green loaded chromaffin cells observed in the absence (upper single cell) and presence (bottom cells) of P-CTX-1B (50 nM) at various times (indicated in minutes). Changes in the fluorescent intensity of sodium-green are displayed in pseudocolour. (Scale bar: 5 µm). (B) The curves show the changes in fluorescence ratio (F/F_0_) of sodium-green as described in the materials and methods section in both control (empty circles) and P-CTX-1B-treated (filled circles) cells. (C) The bar graph shows the peak fluorescent intensity change of sodium-green loaded chromaffin cells in the following conditions: control; P-CTX-1B alone (50 nM); TTX (1 µM) and P-CTX-1B (50 nM); TTX alone (1 µM). Data are expressed as mean±SEM. (Student *t* test, **p<0.01) Scale bar: 5 µm.

One plausible consequence of P-CTX-1B activation of voltage-sensitive Na^+^ channels and membrane depolarization would be a marked increase in intracellular Na^+^. One way to verify this possibility was to measure cytosolic Na^+^ changes induced by P-CTX-1B. Intracellular Na^+^ was monitored in chromaffin cells pre-loaded with the cell-permeant Na^+^-indicator sodium green. Experiments were performed in a Ca^2+^-free medium containing EGTA (2 mM) to avoid activation of the Na^+^/Ca^2+^ exchanger [13]. Exposure of sodium green loaded chromaffin cells to P-CTX-1B (50 nM) resulted in a slow albeit significant increase in intracellular fluorescence intensity that steadily developed continuously for more than 60 min (Figure 1A, B). Under control conditions, the distribution of the fluorescence intensity seemed uniform throughout the cells. Following P-CTX-1B exposure, a slow fluorescence increase was detected throughout the cytoplasm (Figure 1A). The Na^+^ channel blocker tetrodotoxin (TTX, 1 µM) potently inhibited P-CTX-1B-evoked Na^+^ increase (Figure 1C), indicating that voltage-sensitive Na^+^ channel activation is directly involved in this increase. Taken together, these results indicate that P-CTX-1B induced a marked rise of cytosolic Na^+^ in chromaffin cells through an activation of voltage-sensitive Na^+^ channels.

### P-CTX-1B elicits an increase in intracellular Ca^2+^ in chromaffin cells

**Figure 2:**
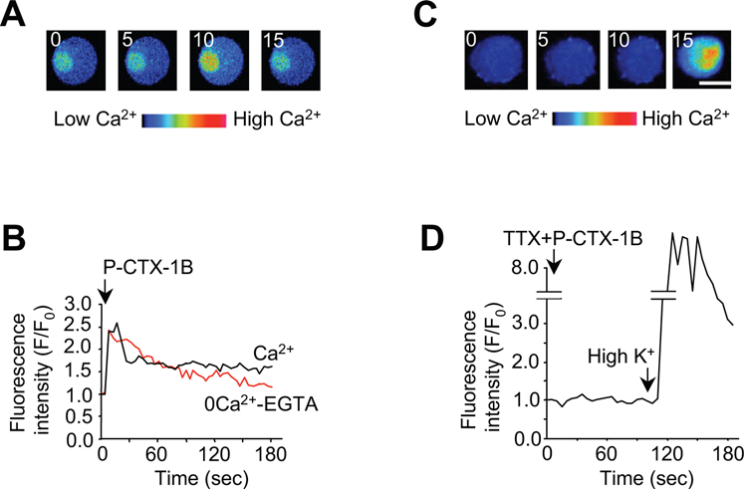
P-CTX-1B produces a transient Na^+^-dependent cytosolic Ca^2+^ increases in bovine chromaffin cells. (A) Optical sections of a confocal-imaged chromaffin cell pre-loaded with fluo-3/AM following the addition of P-CTX-1B (10 nM) to a 2 mM Ca^2+^-containing medium (time in sec indicated in the figure). Scale bar: 10 µm. (B) Relative variation of fluo-3 fluorescence (F/F_0_) determined, as a function of time, in a Ca^2+^-containing medium (black curve, representative of n = 12 cells) or in a Ca^2+^-free medium added with 2 mM EGTA (red curve, representative of n = 6 cells). (C) Same experiment as in A, but chromaffin cells were pre-treated with TTX (1 µM for 15 min) prior to addition of P-CTX-1B to a Ca^2+^-containing medium. High K^+^ was added to the medium after 2 min (last image). Scale bar: 10 µm. (D) Fluorescence variation showing that TTX abolishes the ciguatoxin-induced fluorescence increase. High K^+^-induced depolarization of the cell still produces a robust fluorescence increase.

We next examined whether such long lasting rise in cytosolic Na^+^ could affect intracellular Ca^2+^ level. Confocal microscopy was used to monitor cytosolic Ca^2+^ in bovine chromaffin cells pre-loaded with the cell-permeant Ca^2+^ dye fluo-3/AM. In the presence of 2 mM Ca^2+^, exposure of chromaffin cells to P-CTX-1B (10 nM) resulted in a transient increase in intracellular fluorescence intensity (Figure 2A) followed by a partial recovery during which the fluorescence signal remained higher than the control initial level (Figure 2B, black curve). Interestingly, in the presence of P-CTX-1B, the fluorescence intensity was higher in a localised intracellular region of the cell likely to emanate from the endoplasmic reticulum (ER) (Figure 2A). In the absence of external Ca^2+^, P-CTX-1B was still capable of inducing an increase in fluorescence intensity, but this increase was transitory and returned to basal level (Figure 2B, red curve). These results strongly suggest that at least part of the Ca^2+^ rise elicited by P-CTX-1B originated from intracellular Ca^2+^ stores [14]. Chromaffin cells pre-treatment with TTX (1 µM for 15 min) prevented P-CTX-1B-induced Ca^2+^ influx in a Ca^2+^-containing medium (Figure 2C). Importantly, the same cell was still responsive to high potassium-induced depolarisation known to activate voltage-gated Ca^2+^ channels (Figure 2D). Taken together, these data indicate that, in bovine chromaffin cells, the P-CTX-1B-induced activation of voltage-sensitive Na^+^ channels produces a TTX-sensitive increase in intracellular Ca^2+^ due to both influx of Ca^2+^ into cells and release of Ca^2+^ from internal stores.

### P-CTX-1B triggers Ca^2+^-dependent catecholamine release

**Figure 3:**
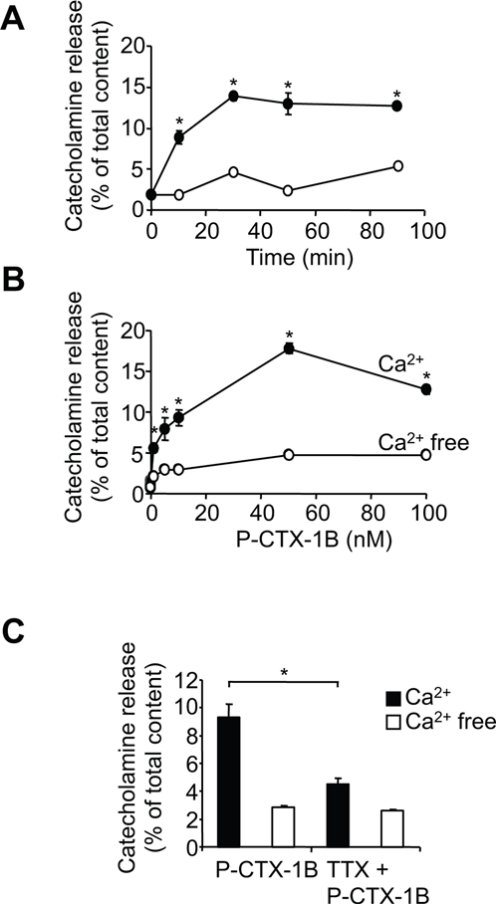
P-CTX-1B triggers a Ca^2+^-dependent catecholamine release via activation of voltage-sensitive Na^+^ channels. (A) Chromaffin cells, bathed in the presence (filled circles) or the absence (empty circles) of 2 mM Ca^2+^ were subjected to P-CTX-1B (10 nM), before removal of an aliquot and assay of its catecholamine content by fluorimetry. Mean amounts of secreted catecholamine (±S.E.M.) are plotted as a percentage of the total content of the cells. P-CTX-1B evokes a Ca^2+^-dependent catecholamine secretion. (B) Chromaffin cells were incubated for 30 min with different concentrations of P-CTX-1B, either with (filled circles) or without (empty circles) external Ca^2+^ (2 mM), before measurement of the amount of catecholamines. The toxin-induced catecholamine secretion is dose-dependent. (C) Amount of catecholamine secreted from chromaffin cells after 30 min exposure to P-CTX-1B (10 nM), in a Ca^2+^-containing (2 mM, black bars) or a Ca^2+^-free (white bars) medium, in the presence or in the absence of TTX (1 µM).

Having demonstrated that P-CTX-1B promotes a significant Ca^2+^ mobilisation in neurosecretory cells, our next step was to investigate whether this signal could be responsible for triggering neuronal secretion. Addition of P-CTX-1B (10 nM) to chromaffin cells in the presence of 2 mM external Ca^2+^ promotes significant catecholamine secretion (Figure 3A). This effect was blocked in the absence of external Ca^2+^ (Figure 3A), indicating that external Ca^2+^ is necessary for P-CTX-1B-induced catecholamine release. The maximal secretion was observed 30 min after P-CTX-1B addition to the medium, indicating that the toxin is a potent albeit relatively slow-acting secretagogue. Concentration-dependency experiments show that the Ca^2+^-dependent effect was maximal at 50 nM (Figure 3B). Finally, TTX (1 µM) pre-incubation blocked P-CTX-1B-induced catecholamine release (Figure 3C), further demonstrating that Na^+^ influx through voltage-sensitive Na^+^ channels was responsible for initiating the secretory effect. Taken together, these data strongly suggest that the activation of voltage-sensitive Na^+^ channels by P-CTX-1B leads to a long-lasting cytoplasmic Ca^2+^ rise, which, in turn, promotes catecholamine release - an effect strictly dependent on the presence of external Ca^2+^.

### P-CTX-1B induces soluble N-ethylmaleimide sensitive factor attachment protein receptor (SNARE)-dependent catecholamine release

**Figure 4:**
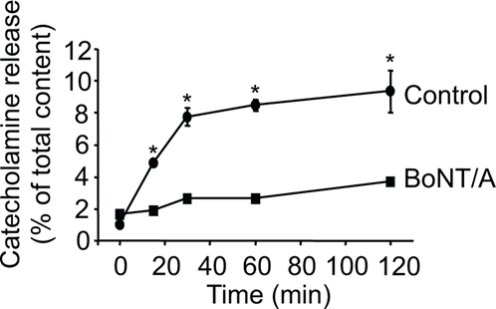
P-CTX-1B triggers SNARE-dependent catecholamine release. Intact chromaffin cells which had been poisoned by prolonged exposure to BoNT/A (66 nM) and control cells were exposed to P-CTX-1B (10 nM) in a Ca^2+^-containing medium (2 mM) before measuring the amount of secreted catecholamines. The P-CTX-1B-induced neurosecretion is impaired by BoNT/A. A representative graph for two experiments carried out in quadruplicate is shown.

To exclude the possibility that cell lysis could promote non-specific catecholamine leakage, we checked whether the release of catecholamine was sensitive to the cleavage of synaptosomal-associated protein of molecular weight 25K (SNAP-25) by Botulinum neurotoxin type-A (BoNT/A). Cultured bovine chromaffin cells were treated *in vitro* by clostridial neurotoxins, using a low ionic strength medium to enable the BoNT internalization for 24 hours [14], [15]. BoNT/A targets and cleaves the synaptosomal-associated protein of molecular weight 25K (SNAP-25), a protein associated predominantly with the plasma membrane, and which is implicated, together with syntaxin and the vesicular protein VAMP/synaptobrevin, in the fusion machinery of catecholamine-containing large dense core granules [16], [17]. Chromaffin cells were washed and subjected to P-CTX-1B in a Ca^2+^-containing medium (2 mM). The catecholamine secretion observed with P-CTX-1B was strongly inhibited by BoNT/A pre-treatment (Figure 4). This result confirmed that the P-CTX-1B-evoked catecholamine secretion occurs through a SNARE-mediated exocytotic process.

### Involvement of intracellular Ca^2+^ stores in P-CTX-1B-induced catecholamine release

**Figure 5:**
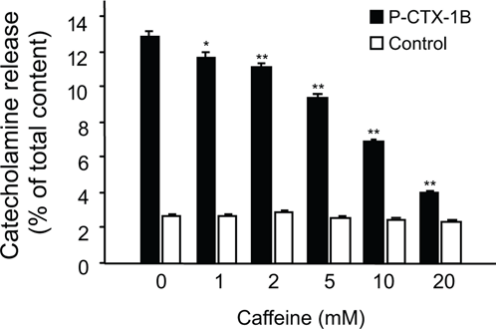
Implication of Ca^2+^ stores on ciguatoxin-evoked release of catecholamines. Chromaffin cells were pre-treated (20 min) with increasing concentrations of caffeine and subsequently exposed (black bars) or not (white bars) to P-CTX-1B (10 nM), before measuring the amount of secreted catecholamines. The P-CTX-1B-induced catecholamine release is decreased following depletion of internal caffeine-sensitive Ca^2+^ stores. A representative graph for three experiments carried out in quadruplicate is shown.

In view of our previous evidence for a role of intracellular Ca^2+^ stores in P-CTX-1B-evoked Ca^2+^ rise (see Figure 2), we examined the possible role of caffeine-sensitive Ca^2+^ stores in mediating P-CTX-1B-evoked catecholamine release. Pre-incubation of chromaffin cells with increasing concentrations of caffeine in a Ca^2+^-containing medium, prior to P-CTX-1B stimulation, resulted in a significant inhibition of P-CTX-1B-induced catecholamine release (Figure 5, black bars). Importantly, in bovine chromaffin cells, caffeine alone did not promote catecholamine secretion (Figure 5, white bars), as reported previously [14], [18], [19]. It should be noted that caffeine induces an intracellular Ca^2+^ increase in bovine chromaffin cells that corresponds to a Ca^2+^ release from internal stores of the ER [19]. Our result strongly suggests that internal caffeine-sensitive Ca^2+^ stores are indeed required for mediating the secretagogue effect of P-CTX-1B.

**Figure 6:**
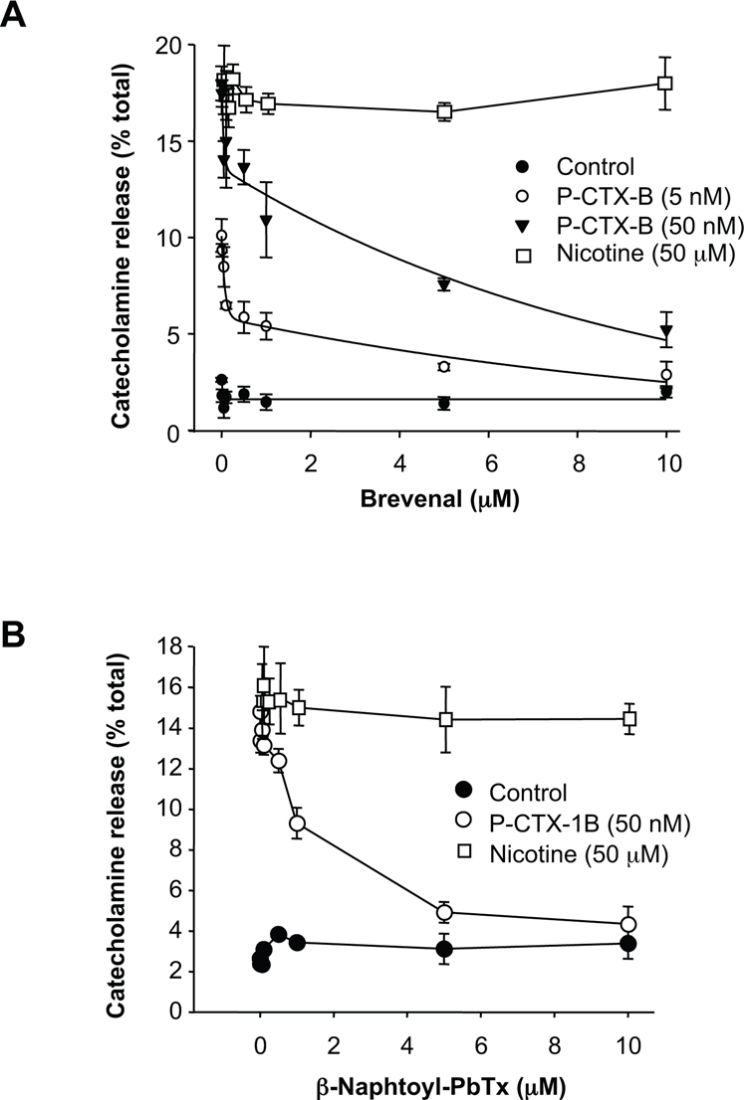
Brevenal and β-naphtoyl-brevetoxin abolishes ciguatoxin-evoked release of catecholamines. Chromaffin cells were pre-treated (15 min) with increasing concentrations of either brevenal (A) or β-naphtoyl-brevetoxin (B) and subsequently exposed to indicated concentrations of P-CTX-1B, nicotine or vehicle alone as indicated in the figure, before measuring the amount of secreted catecholamines. Data are expressed as mean±SEM. Only P-CTX-1B-induced catecholamine release is blocked by brevenal and β-naphtoyl-brevetoxin pre-treatments. Representative graphs for three experiments carried out in octoplicate are shown.

Having established bovine chromaffin cells as a good model to investigate the mechanism underpinning P-CTX-1B neurosecretory effect, we explored ways to block this secretagogue activity. Brevetoxin and P-CTX-1B share an identical binding site on voltage-sensitive Na^+^ channels (VSSC), called receptor-site 5 [20], [21]. In view of the recent discovery of brevenal, a natural compound from *Karenia brevis* found to selectively displace brevetoxin from site 5 of the VSSC [22], we hypothesised that brevenal could also act as a potent inhibitor of P-CTX-1B stimulatory action. Pretreatment of chromaffin cells with increasing concentrations of brevenal (in µM: 0, 0.01, 0.05, 0.1, 0.5, 1.5, 10) did not have any noticeable effect on catecholamine secretion (Figure 6A). However, P-CTX-1B (10 nM) stimulatory effect was inhibited in a dose-dependent manner by brevenal (Figure 6A). This significant inhibitory effect exhibited an initial phase of inhibition for nanomolar concentrations of brevenal, followed by a less potent inhibitory phase for micromolar concentrations (IC_50_∼2 µM). This biphasic effect was accentuated by raising P-CTX-1B concentration to 50 nM (Figure 6A). The inhibitory effect of brevenal was reversible by simple washout (data not shown). Importantly, brevenal did not significantly affect nicotine-induced catecholamine secretion (Figure 6A). This demonstrates that the ability of nicotinic acetylcholine receptors to respond to nicotine stimulation was not modified by brevenal in chromaffin cells. β-Naphtoyl-brevetoxin, another competitive inhibitor of brevetoxin binding on VSSC was also tested in our system. Similarly, β-naphtoyl-brevetoxin prevented P-CTX-1B-induced catecholamine secretion without interfering with nicotine secretagogue activity (Figure 6B).

## Discussion

Voltage-sensitive Na^+^ channels are transmembrane proteins responsible for the depolarising phase of action potentials in excitable cells [21]. These channels are targeted by several neurotoxins, which alter their biophysical properties, physiological regulation and thus the related functional activity. Our investigation reveals that P-CTX-1B promotes a TTX-sensitive long-lasting cytosolic Na^+^ increase in bovine chromaffin cells. This, in turn, triggers an intracellular Ca^2+^ increase responsible for eliciting catecholamine secretion from chromaffin cells. This secretion is strictly dependent on external Ca^2+^, suggesting that a Ca^2+^ influx is required, and appears to be mediated by Ca^2+^ stores, as it is sensitive to caffeine treatment. P-CTX-1B is shown herein to be a potent albeit slow-acting secretagogue for neuroendocrine cells. The induced catecholamine secretion results from a Na^+^-mediated Ca^2+^ entry through regulated exocytosis, as revealed by its potent inhibition by BoNT/A. Finally, we show that both brevenal and β-naphtoyl-brevetoxin prevent P-CTX-1B-induced catecholamine secretion – a finding with ramification for future treatment of the widespread human disease ciguatera.

### Deciphering the various steps leading to P-CTX-1B-induced neuronal secretion: initial Na^+^ influx

Ciguatoxins bind to voltage-sensitive Na^+^ channels with high affinity [4], [5], [23]. These cyclic polyethers compounds modify Na^+^ current by shifting the voltage-dependency of Na^+^ channel activation towards more negative values, and by partly suppressing their inactivation [8], [24]. There are a variety of functional consequences to promoting such Na^+^ influx in excitable cells: trains of spontaneous action potentials, volume increase of nodal region of frog myelinated nerve fibres [8], [25], [26], enhancement of neurotransmitter release, volume increase of motor nerve terminals at the frog neuromuscular junction [9], [25], spontaneous activity in neuroblastoma cells [23], activation of the Na^+^/Ca^2+^ exchanger in the reversed mode followed by neurotransmitter release from synaptosomes [13], and Ca^2+^ mobilisation in neuroblastoma cells [12]. It was therefore important to clarify the effects of such a Na^+^ channel activator on intracellular concentrations of Na^+^ and Ca^2+^ and neurotransmitter release. In the presence of ciguatoxins, large quantities of Na^+^ are believed to flow into cells during the depolarizing phase of spontaneous and/or repetitive action potentials as a result of the partial inhibition of voltage-sensitive Na^+^ channel inactivation [8], [27], [28]. Such high Na^+^ concentration is compensated by a water influx, that can be reversed or prevented using the hyperosmotic agent D-mannitol [8], [26]. Our results differ from those of Bidard *et al.* [23], in which measurements of ^22^Na^+^ in neuroblastoma cells indicated that ciguatoxin could only promote detectable ^22^Na^+^ increase only if it acts in synergy with other voltage-sensitive Na^+^ activators, such as veratridine or batrachotoxin [23]. The requirement for co-activation of voltage-sensitive Na^+^ channels in promoting intracellular Na^+^ increase was confirmed with the use of brevetoxin-3 combined to veratridine in chromaffin cells [29]. Likewise, cyanobacterial antillatoxin was reported to induce a Na^+^ increase in neuronal cells through direct activation of voltage-sensitive sodium channels at site 4 [30]. Our study, using confocal imaging techniques, reveals a slow albeit robust Na^+^ influx in response to P-CTX-1B alone without requiring the synergistic action of another activator. This Na^+^ increase was almost totally inhibited by TTX pre-treatment, providing compelling evidence for the implication of voltage-sensitive Na^+^ channels in this effect as also shown in neuroblastoma cells [23]. These experiments were conducted in a Ca^2+^-free medium supplemented with EGTA to avoid the Na^+^/Ca^2+^ exchange system to be activated in the reversed mode, as it has been demonstrated previously [13]. This apparent discrepancy could be explained by the sensitivity of the two methods used and the relatively short duration of the experimental design used in Bidard *et al*. Indeed, initial rate of ^22^Na^+^ uptake was only determined after 1 min during which little Na^+^ influx was observed in our hands [23].

### P-CTX-1B promotes Ca^2+^ influx coupled with Ca^2+^ release from the intracellular Ca^2+^ stores

In chromaffin cells, P-CTX-1B-induced activation of voltage-sensitive Na^+^ channels produced a rise in cytosolic Ca^2+^ detected either in a Ca^2+^-containing or a Ca^2+^-free medium. The opening of voltage-sensitive Na^+^ channels has been reported to lead to membrane depolarization and the consequent activation of voltage-gated Ca^2+^ channels [31] and to the activation of reverse Na^+^/Ca^2+^ exchangers which extrudes Na^+^ under pathophysiological conditions [32]. Alternatively, the CTX-induced sodium influx could simply reduce the sodium gradient that normally drives the removal in intracellular Ca^2+^ via the Na^+^/Ca^2+^ exchanger, allowing accumulation of Ca^2+^ in the stores. The combination of these effects may explain why a Ca^2+^ increase was recorded following the addition of P-CTX-1B. This transient Ca^2+^ rise was TTX-sensitive and spatially restricted. This type of localized Ca^2+^ increase is classical in chromaffin cells where Ca^2+^ stores such as those of the endoplasmic reticulum are segregated in specialized region [33]. Therefore our results show that cytosolic Ca^2+^ transient increase results from the combination of a Ca^2+^ influx into the cells and a Ca^2+^ release from internal Ca^2+^ stores, as previously reported in chromaffin cells [14] and neuroblastoma cells [12]. Following the intracellular rise in Ca^2+^ level, the return to basal level was not achieved in the presence of P-CTX-1B. This suggests that P-CTX-1B initially induces a Ca^2+^ influx closely followed by a Ca^2+^ release from intracellular Ca^2+^ stores necessary to maintain the level of secretion, as suggested by the inhibitory effect obtained with caffeine pre-treatment. Importantly, an alternative source of Ca^2+^ entry through a store-operated mechanism could be involved, as to help replenish intracellular Ca^2+^ stores in response to their full or partially Ca^2+^ depletion [34]. We demonstrate here that P-CTX-1B-induced activation of voltage-sensitive Na^+^ channels produced a rise in cytosolic Ca^2+^ in chromaffin cells detected either in a Ca^2+^-containing or a Ca^2+^-free medium, suggesting that Na^+^ influx may directly couple to intracellular calcium release.

### P-CTX-1B promotes the SNARE-dependent exocytosis of catecholamine-containing secretory vesicles

In this study, we show that P-CTX-1B is a potent secretagogue for chromaffin cells which is highly dependent on external Ca^2+^. This suggests that the activation of voltage-sensitive Na^+^ channels produces a Ca^2+^ influx necessary for triggering neurosecretion. The evoked catecholamine secretion results from the initial activation of voltage-sensitive Na^+^ channels by P-CTX-1B, since it was abolished by TTX pre-treatment. This catecholamine secretion occurs through regulated exocytosis of catecholamine containing large dense core vesicles, as revealed by its sensitivity to the cleavage of SNAP-25 by BoNT/A.

Finally, the secretagogue activity of P-CTX-1B requires intracellular Ca^2+^ stores, since pre-emptying the stores with caffeine significantly decreased its stimulatory effect. Bearing in mind that direct stimulation of Ca^2+^ stores is unable to trigger catecholamine secretion [35], our result demonstrate that Ca^2+^ from internal stores is necessary to potentiate catecholamine secretion probably by inducing a Ca^2+^ influx through a store-operated entry pathway [36].

### Brevenal and β-naphtoyl-brevetoxin prevent P-CTX-1B stimulatory effect without affecting nicotinic response

Our result shows that both brevenal and β-naphtoyl-brevetoxin are capable of blocking the stimulatory effect of P-CTX-1B, at physiologically relevant concentrations, demonstrating that both compounds act as functional antagonists for ciguatoxin. Brevenal is a non-toxic 8-fused ring polyether natural compound produced by *K. brevis* that was shown to successfully displace [^3^H]-PbTx-3 from receptor site 5 of VSSC [37]. Moreover, brevenal and gambierol have recently been shown to be potent antagonists of PbTx-2-induced Ca^2+^ influx in neurons [38]. The relevance of the competitive nature of brevenal towards brevetoxin has been argued on the basis of structural differences [39]. However, brevenal has been demonstrated to be a potent functional antagonist that confers protection from brevetoxin-induced lethality in fish [37], [40] and in an asthma model in sheep [41]. Brevenal is a lead compound for the development of treatment for cystic fibrosis and neurotoxic shellfish poisoning. Our results strongly suggest that brevenal could be a molecule of choice for the potential treatment of ciguatera.

In conclusion, our results provide conclusive evidence that, following ciguatoxin treatment, the slow increase in intracellular Na^+^ through VSSC leads to an influx of Ca^2+^ relayed by activation of a Ca^2+^-induced Ca^2+^ release which significantly contributes to the secretion of catecholamines. Ciguatera is a dangerous condition especially considering that, with the global tropical fisheries markets, symptoms of ciguatera are starting to appear well outside the endemic regions, eg. in Europe [3]. There is no known cure for ciguatera condition, although mannitol can provide limited symptomatic relief (1). A full understanding of the molecular mechanisms initiated by ciguatoxin binding to voltage-sensitive Na^+^ channels through to the release of neurotransmitter is necessary to develop new therapies for this type of channelopathy. Our study demonstrates that brevenal, a natural compound extracted from *Karenia brevis*, can potently inhibit ciguatoxin's stimulatory effect on exocytosis therefore providing the basis of the first potential specific treatment of ciguatera.

## Materials and Methods

### Cell culture

Bovine chromaffin cells were prepared from adrenal glands and maintained as primary cultures, following procedures previously described [15]. Within 2-3 days, cells were incubated in the presence or in the absence of botulinum type A neurotoxin (BoNT/A, 66 nM) in a low ionic strength buffer (37°C, 24 hours) to facilitate BoNT/A uptake [42]. Chromaffin cells were then returned to their original medium for 24 hours before starting experiments.

### Drugs

P-CTX-1B was extracted and highly purified from the Pacific moray-eel *Gymnothorax javanicus* [43], [44]. The dry toxic extracts were dissolved in ethanol, and the solution was divided into several samples. The ethanol was then evaporated. Samples were kept dry at −20°C and diluted with external solutions immediately before experiments. Brevenal and PbTx-2 were isolated from cultures of *K. brevis* (Wilson Clone) [40] and aliquoted into 0.5 mg vials and stored dry at −20°C, β-naphtoyl-brevetoxin was synthesized from PbTx-2 [45], aliquoted into 0.5 mg vials and stored dry at −20°C. Tetrodotoxin (TTX) was purchased from Sigma (St Louis, MO, USA); Fluo-3/AM and sodium green/AM were purchased from Molecular Probes (Invitrogen), caffeine from Sigma Chemical Co. (Dorset, UK). BoNT/A was purified as previously described [46], and stock solutions were stored in 150 mM NaCl and 10 mM 4-(2-hydroxyethyl)-1-piperazineethanesulfonic acid ) (HEPES) buffer (pH 7.4) at −20°C.

### Confocal microscopy

Chromaffin cells were loaded by incubation (30–45 min, room temperature) with either fluo-3/AM or sodium green/AM in Krebs-Ringer solution. After washing the cells with a dye-free solution, the coverslips were placed on the stage of the microscope and imaged by confocal microscopy. A Sarastro 2000 or a Zeiss 510 META confocal laser scanning microscope (Molecular Dynamics, USA) was used. The aperture setting of the confocal pinhole was 100 µm. Cells were either visualized using a 40× or a 60× water immersion objective (Zeiss 0.75 and 0.9 numerical aperture respectively). The 488 nm band of the Argon ion laser was used for excitation and the emitted light was collected through a 510 nm long-pass. All experiments were performed at room temperature and time series of cell confocal sections were acquired (using a time-lapse mode) with a frame interval of 5 s for fluo-3/AM and 5 min for sodium green/AM. Sections were collected using a standard scanning mode format of 512×512 pixels. The fluo-3/AM or sodium green/AM loaded cells were presented using a pseudocolor scale. The index of fluorescence variation was calculated and presented as F/F_0_, where F_0_ is the initial cell fluorescence and F the monitored cell fluorescence.

### Measurement of catecholamine secretion

The catecholamine secretion was quantified as previously described [14]. Briefly, chromaffin cells were washed once with HEPES-buffered saline solution containing (in mM) 145 NaCl, 5 KCl, 1.2 NaH_2_PO_4_, 10 glucose, 20 HEPES (pH 7.4), and processed in quadruplicate. Aliquots (200 µl) of the medium were removed at the end of each experiment, and cells were lysed with Triton x-100 (1%, v/v). Both set of samples were assayed fluorimetrically for catecholamine content. Amounts released were expressed as a percent of the total content of catecholamines present in the cells [18]. Data were representative of experiments carried out in quadruplicate and performed at least 3 times.

### Statistical analysis

Data analysis was carried out using Student's *t* test. All experiments were performed at least 3 times. Values were expressed as mean±SEM and data were considered significant at *p<0.05 and **p<0.01.
